# COVID-19 and Cancer Patients in the Second Year of the Pandemic: Investigating Treatment Impact, Information Sources, and COVID-19-Related Knowledge, Attitudes and Practices

**DOI:** 10.3390/curroncol29110701

**Published:** 2022-11-18

**Authors:** Mohamed A. Ugas, Lisa Avery, Yanning Wang, Alejandro Berlin, Meredith E. Giuliani, Monika Krzyzanowska, Tina J. Papadakos, Naa Kwarley (Linda) Quartey, Diana Samoil, Janet K. Papadakos

**Affiliations:** 1 Cancer Health Literacy Research Centre, Cancer Education, Princess Margaret Cancer Centre, Toronto, ON M5G 2N2, Canada; 2 Biostatistics Department, Princess Margaret Cancer Centre, Toronto, ON M5G 2C1, Canada; 3 Institute of Health Policy, Management and Evaluation, Dalla Lana School of Public Health, University of Toronto, Toronto, ON M5T 3M6, Canada; 4 Department of Radiation Oncology, University of Toronto, Toronto, ON M5T 1P5, Canada; 5 Radiation Medicine Program, Princess Margaret Cancer Centre, Toronto, ON M5G 2C1, Canada; 6 Techna Institute, University Health Network, Toronto, ON M5G 2C4, Canada; 7 The Institute for Education Research (TIER), University Health Network, Toronto, ON M5T 1V4, Canada; 8 Division of Medical Oncology and Hematology, Princess Margaret Cancer Centre, Toronto, ON M5G 2C1, Canada; 9 Division of Medical Oncology, University of Toronto, Toronto, ON M5S 3H2, Canada

**Keywords:** COVID-19, coronavirus, cancer, health literacy, vaccination, knowledge, attitudes, practices

## Abstract

Background: The novel coronavirus that has triggered the present COVID-19 pandemic continues to spread globally, resulting in widespread morbidity and mortality. Patients with cancer remain one of the most vulnerable subsets of the population to the disease. This study examined the effects of the pandemic on cancer patients’ treatment, psychology, knowledge, attitudes, and practices. Methods: A survey was emailed to 9861 patients at a cancer centre in Toronto, Canada. Descriptive results were summarized. Qualitative feedback was coded and summarized. Regression modelling was used to explore factors associated with patient psychological well-being, knowledge, attitudes, and practices. Results: A total of 1760 surveys were completed, with a response rate of 17.8%. Most participants did not experience any pandemic-related treatment delays, and vaccination rates were high. Participants who identified themselves as non-white (OR 3.30, CI: 1.30–5.30; *p* ≤ 0.001), and those who referred to journal articles for information (*p* = 0.002) reported higher psychological impact scores. There were no significant predictors of whether participants would use personal protective equipment when leaving their homes or whether they would go to crowded places. Discussion: This study provides another snapshot of cancer patients perceptions and needs during the COVID-19 pandemic.

## 1. Introduction

The novel coronavirus that has triggered the present COVID-19 pandemic continues to spread globally, resulting in widespread morbidity and mortality [1]. While the emergence of new methods of prevention and treatment, including several highly efficacious vaccines and antiviral therapies, have offered tools to combat the disease, the emergence of more virulent and transmissible variants, combined with the relaxation of non-pharmaceutical interventions, has resulted in SARS-CoV2 remaining a major cause of death internationally, with the World Health Organization estimating that nearly 6.5 million people have succumbed to the virus, a figure that likely undercounts the true toll when excess deaths are considered [1]. 

Patients with cancer remain one of the most vulnerable subsets of the population to COVID-19 infection. They are also overrepresented in two other at-risk groups: the elderly and the immunocompromised. As such, the impact of the pandemic on these sections of the population is an important area of study. Restrictions placed on the recipients of healthcare services in the interest of infection control may result in hindering access, while patients themselves may delay treatment to protect themselves, especially considering the complex nature of cancer treatment [2,3]. Early studies have found that cancer patients’ attitudes to lockdown measures were positive, and only those needing acute care continued to attend hospitals regularly [4]. Other studies, however, have found that patients with cancer have suffered from low quality of life because of lockdown measures and have found telehealth to be inadequate to meet their care needs [5,6].

As an at-risk population, the perceptions of patients with cancer are important as they may shape practices that contribute to their safety. They can also provide information on whether healthcare systems are meeting the needs of the most vulnerable sections of the population during a public health crisis. Understanding these attitudes and perceptions may prove useful in dealing with the mental health ramifications of the public health situation and prolonged isolation. Greater levels of risk perception among patients with cancer during the outbreak have been associated with more distress [7]. Patients have also cited social isolation as the most difficult aspect of dealing with cancer during the pandemic [3].

This study seeks to evaluate the impact of COVID-19 on the psychological well-being and access to cancer health care services of patients with cancer at the end of the fourth wave of the pandemic. It further seeks to investigate their knowledge, attitudes, and practices regarding COVID-19, as well as the sources of information they have consumed. This study seeks to identify predictors and to contrast the results with those found in the first iteration of this survey, administered in the summer 2020 (between the first and second waves [8]), particularly considering the availability of vaccines against the coronavirus. 

## 2. Materials and Methods

The study replicated the methodology used by the authors in an earlier paper [8] by employing a cross-sectional design, with a survey administered to patient participants recruited from a large academic cancer centre in Toronto, Canada. The results of the survey were then used to model relationships between various patient characteristics and outcomes, including knowledge, psychological impact, use of preventative measures, and media sources consumed. Patients were eligible for the survey if they were at least 18 years old, were able to read and write in English, and were currently receiving cancer care. Ethics approval was obtained, and consent was implied by survey completion. A link was sent out with email addresses obtained from the hospital’s Virtual Care Management System, and patients were invited to complete the anonymous survey on the LimeSurvey online platform [9]. The survey was first sent out on 21 October 2021. A reminder invitation was sent out on 28 October 2021, and a final notice for participants to complete the survey was issued on 4 November 2021. 

### 2.1. Survey Structure

The survey comprised ten sections (Supplementary File). Section S1 (demographics) asked participants about their age, gender, income, education, race, living arrangements, cancer type and other personal characteristics. Health literacy was assessed in this section as well, using a validated, single-item screening tool that asks the question, “Are you comfortable filling out medical forms on your own?”, adapted from Chew and colleagues [10]. To determine the extent of their exposure risk to the virus, participants were asked if they considered themselves essential workers, with grocery store workers, bus drivers, and healthcare workers given as examples. Section S2 (treatment impact and concerns) focused on impacts on cancer treatments due to the pandemic. For example, participants were asked if their treatment was delayed, and if so, for how long. They were also asked to rate their concern over whether the pandemic might adversely affect their prognosis (referred to as cancer worry) and whether they feared infection at the hospital, both using five-point Likert scales. Section S3 (COVID-19 information sources and quality) asked participants to select from a list the sources they consult for information about COVID-19 and to rate the frequency with which they sought information from the source. They were also asked to evaluate the quality of the different information sources in the context of COVID-19. Section S4 (psychological impact) asked participants to indicate the psychological impact of COVID-19 by rating their agreement on statements concerning fears and anxieties with respect to the pandemic. Participants were asked if they felt isolated, had difficulty sleeping or focusing on tasks, had feelings of anxiety and irritability, and about fears that they themselves or their loved ones might contract the virus. Section S5 (knowledge) asked participants “True” or “False” questions to gauge their knowledge of the symptoms of COVID-19, risk factors, transmission, and prevention, based on what was known about the virus at the time of survey deployment. Section S6 (practices) asked participants to answer “Yes” or “No” to whether participants adhered to best practices aimed at preventing the spread of COVID-19 including hand washing, the wearing of masks, and social distancing measures. Section S7 (attitudes) included questions about participant’s attitudes to the pandemic, using a five-point scale to determine if they thought it could be controlled, whether the cancer centre was doing an adequate job in response, and whether they were confident they could avoid infection personally. Section S8 concerned vaccine uptake and attitudes. Participants were asked whether they received the vaccine, which version of the vaccine they received, what their motivations for doing or not doing so were, and what hurdles they may have faced. These questions were adapted from the CDC vaccine question bank [11]. Section S9 (discrimination) asked the participants 6 questions related to racism and the pandemic including whether one should avoid people from Brazil or India (countries that had, at the time of the survey, been recently hit by devastating waves of the virus), whether they had witnessed or were the target of a racist incident, and the nature of that incident. Section S10 (most needed information) comprised two open-ended questions asking participants to indicate their most pressing information needs and asking for any additional comments. Note that to promote flow in the survey, knowledge, attitudes, and practices appear in order of knowledge, practices, and attitudes (Supplementary File). 

### 2.2. Statistical Analysis

Descriptive statistics were used to summarize the results. To investigate factors associated with cancer worry, knowledge, attitudes and practices, multivariable models were fit with candidate predictor variables informed by both a priori hypotheses of which variables would be significant and univariate regression analyses. To reduce the likelihood of type I errors, Bonferroni-corrections were applied to univariate regressions for each outcome to identify significant predictors. For continuous outcomes (cancer worry, COVID-19 knowledge, COVID-19-related attitudes, engagement in preventive practices), linear regressions were modelled, and model assumptions were checked using plots of standardized residuals and normal Q-Q plots. 

For the regression analyses, variables were coded as follows: Education: low (some high school and grade school), medium (some college and college), and high (postgraduate); Race: white versus non-white; Income: <$40 k, $40–60 k, $60–100 k, and greater than USD100 k; Cancer Type: blood cancers versus others. Continuous measures of cancer worry, preventative practices, psychological impact and knowledge were computed by summing the relevant survey questions and tested for internal consistency.

The most used information sources were identified by the percentage of participants who answered “Often” or “Always” in reporting their usage. To determine factors associated with use of information sources, multivariable ordinal regression models were fit. The assumption of proportional odds was assessed visually by comparing logit spacing across categories in the manner described by Harrell [12]. The Holm-adjusted *p*-value was calculated to control for multiple testing and holds the type-I error rate for each analysis at 5%. The R statistical programming language was used for all quantitative analysis.

### 2.3. Open-Ended Responses 

Data derived from the two open-ended questions at the end of the survey were organized and analyzed using the qualitative data software program NVivo [13]. The responses were categorized thematically, using inductive coding, and summarized with representative quotations. 

## 3. Results

Surveys were emailed to patients in the fall of 2021 with responses completed between 21 October and 12 December. Of the 9861 that were sent out, 1760 were completed for a response rate of 17.8%. 

### 3.1. Descriptive Statistics

The median age of participants was 64 years (range: 19–100 years of age). A total of 51% identified themselves as female and 49% as male. No participants identified as non-binary genders. Nearly two-thirds (62%) were born in Canada, and more than three quarters (76%) spoke English as their first language. Most participants (94%) answered in the affirmative to the health literacy metric regarding whether they were able to fill out forms in English, and 62% reported a medium level (some college and college) of educational attainment. Overall, 40% reported working full or part-time, and of those, 74% identified themselves as essential workers. The majority (81%) were suffering from solid-tumor cancers, with most (60%) in follow-up after the conclusion of their treatment. At the time of the survey, 60% were in follow-up post-treatment, and 17% were in active treatment. Nearly two-thirds of participants (63%) reported not experiencing any treatment delays (Table 1). Of those that did experience treatment delays, 27 (2%) experienced delays less than 2 weeks long, and 86 (5%) were delayed for between 2 weeks and 3 months. With respect to modality, 59% of participants had their appointments made virtual (Table 2). 

A plurality of participants (29%) were neutral on whether the pandemic would make it difficult to get cancer care in the future and whether they would experience complications due to the pandemic (33%). Thirty percent disagreed that their cancer would return and not be detected or managed properly due to the pandemic. Sixty-nine percent disagreed or strongly disagreed that they would contract the coronavirus by coming to the cancer centre (Table 3).

Information sources varied among participants with most more commonly relying on television (24% = always) and the public health press releases (36% = always) for information about COVID-19, compared to social media (2% = always). Perceptions of the quality of each source were mixed, with public health department and press releases (36%) along with cancer centre resources (32%) being rated as “excellent” most often (Table 4). The median psychological impact score was 65 out of 100 (with a range of 20–100), indicating moderate impact of the pandemic on mental health (Table 5). Knowledge scores were high, with between 88–98% answering correctly for each question except for a single item regarding how common it is for cold-like symptoms to manifest with COVID-19 and half answered incorrectly (Table 6). 

Psychology Score

- Mean (SD): 63.7 (16.2)
- Median (Min, Max): 65.0 (20.0–100.0)

Preventative practices for infection control were similarly very high among participants, ranging from 89–99% for each except for quarantining, only 69% of which reported doing (Table 7). Attitudes regarding the pandemic were optimistic, with 59% agreeing that the pandemic could be controlled successfully, and 43% strongly agreeing that they could protect themselves from COVID-19 (Table 8). Two-hundred and fifty participants did not answer whether they were vaccinated. Of those that did, 97% received at least one dose, and of those that did not (*N* = 46), 53 were not planning on doing so. The biggest motivating factor for getting vaccinated was to protect one’s health (88%) and the least commonly cited factor was the encouragement of others (7%; see Table 9). Most participants (48%) agreed that one should avoid people where COVID-19 case numbers are high (Table 10). 

### 3.2. Multi-Variable Regression

The preventative practice, cancer worry, and knowledge metrics all scored poorly on internal consistency and were excluded from the multivariable analysis. 

Participants who identified themselves as non-white (OR 3.30; *p* ≤0.001), and those who referred to journal articles for information, reported higher psychological impact scores (Table 11). A larger proportion of non-white participants did not answer the questions regarding psychological impact. Participants who used public health department and press releases or television as their sources for COVID-19 information, were more likely to be vaccinated, as were those with household incomes of greater than USD 40,000 and older patients, although this effect was marginal (OR 1.03; *p* = 0.035). Older participants and non-white participants were less likely to answer the vaccination question (Table 12).

Older participants were more likely to use television as a source of information (OR 1.02; *p* ≤ 0.001) than younger participants. Participants with high education were less likely to use television (OR 0.54; *p* ≤ 0.001), while participants who rated the quality of television information highly were more likely. Older age, high education and perceived quality were associated with use of print media. The use of friends and family was associated with not speaking English at home (OR 1.51; *p* ≤ 0.001) while working full or part-time was associated with the use of work colleagues as a source of information. Older participants reported being less likely to use social media (OR 0.96 *p* ≤ 0.001) or search engines (OR 0.98 *p* ≤ 0.001). Non-English speakers were less likely to answer questions on information sources (Table 12).

Age, although statistically significant (*p* ≤ 0.001) has a small effect on the concern that a recurrence of cancer will not be detected or managed properly due to COVID-19, with the odds of increased worry decreasing minimally with increased age (OR 0.98). Female participants tend to worry more than males (OR 1.37; *p* = 0.004), and non-white participants worry more than white participants (OR 1.43; *p* = 0.01). Participants who refer to public health literature for their information tend to worry less than those who never or rarely refer to public health resources. Non-white participants worry more about contracting COVID-19 at the hospital compared to white participants (OR 1.53; *p* = 0.03), as were participants who never use television as a source for COVID-19 information, compared to those that always do (*p* = 0.04). Patients who refer to public health literature as a source for COVID-19 information tend to worry more than those who never or rarely refer to these resources about contracting COVID-19 at the hospital (*p* = 0.02). There were no significant predictors of whether participants would use personal protective equipment when leaving their homes or whether they would go to crowded places (Table 12). 

### 3.3. Open-Ended Comments

In written feedback, participants primarily inquired about how they could obtain actionable information (primarily about vaccine boosters), how to maintain their safety, offered further context on the impact of the pandemic on their treatment/care, and expressed gratitude to the hospital and their healthcare providers. Participants also offered criticisms of the hospital (mostly concerning restrictions on visitors/caregivers), voiced support or opposition to vaccinations/vaccine mandates, and commented on the public health situation and the government’s handling of it. 

## 4. Discussion 

Our findings indicate that more than 20 months since the emergence of COVID-19, patients with cancer remain concerned about the pandemic, and are continuing to exhibit accurate knowledge about the disease, and undertake preventative measures, now including vaccine uptake. Rates of vaccination were high, while treatment continued with limited disruption for most patients despite the high disease burden in the population due to the spread of easily transmissible variants of concern. 

At the time of the survey, Canada was at the tail-end of the Delta variant driven fourth wave of the virus. While the variant had devastated other parts of the world and other regions of Canada, Ontario was not as severely affected [14]. Vaccination rates in the country were relatively high, and mandates had been introduced to compel healthcare workers to get vaccinated [15]. Breakthrough cases were not as common, and while news of the Omicron variant would have reached participants completing the survey in late November and early December, early reports stressed that the variant was “mild” relative to its predecessors. These factors may have combined to produce optimism among participants that the outbreak was beginning to taper off. Certain restrictions had been eased by this point, such as those on caregivers accompanying patients to the hospital, which had been identified as a particular source of displeasure among patients who relied on them for support [3]. The wide use of virtual care, however, has been welcomed by some patients with cancer, who point to reduced cost and the diminished reliance on caregivers to assist with an in-person visit [16], with both caregivers and patients reporting satisfaction with quality of care [17]. 

As a group, the participants displayed continued adherence to preventative measures despite the increasing risk of pandemic fatigue [18] as well as the increasing laxity associated with higher vaccine uptake [19]. Consistent with other findings, female participants reported greater perceived risk to themselves [20]; however, unlike the previous survey and other studies, they did not report greater levels of preventative practices [8,21]. This may be due to the high levels of precautions among patients with cancer in recognition of the higher risk they face. Similarly, non-white participants also expressed greater fears of the contracting the virus and its impact on their treatments. This may reflect the greater rates of COVID-19 infection witnessed among racialized groups in Toronto relative to their white counterparts [22]. This has been attributed to higher rates of comorbidities, as well as types of housing and areas of employment that undermine efforts at social distancing [23]. While this version of the survey collected data on sexual orientation, the numbers were not substantial enough to carry out analysis comparing different groups, although research indicates that belonging to non-heterosexual orientations may be related to greater adherence to precautionary measures [20].

Compared to the previous iteration of this survey, there were slight reductions in the psychological impact of COVID-19 on patients. This may be further evidence of the adjustment of perceptions to COVID-19 as well as a degree of survival bias in our sample. This may also be reflected in the responses to participants’ perceptions of personal risk and the threat posed to their treatment by COVID-19 with, for instance, the proportion of those strongly disagreeing that they would contract the virus at the hospital more than doubling. A shifting of expectations may also be the cause of these changing perceptions, such as the increasingly held view that the virus will become endemic in the population. For instance, while more participants in this survey believe the pandemic can be successfully controlled, fewer believe they themselves can avoid infection, compared to the survey administered in 2020 [8]. This likely stems from the increased protection offered by vaccines and the availability of treatments that reduce the risk for severe outcomes among those who contract the virus. 

Our findings are complicated by non-response to some survey items. For example, 14% of participants declined to report their vaccination status. At the time of the survey, as mentioned, governments began instituting mandates to encourage vaccination, resulting in a polarized social debate on these measures [24]. This may explain the reluctance to share that information among participants, perhaps even among those who did obtain and support vaccination [25]. Early rates of hesitancy in the population had subsided at that point, as more than three quarters of the population had begun the initial vaccine series; however, misinformation about COVID-19 vaccines remained ubiquitous [26]. A large majority of participants also declined to identify whether they were essential workers or not. This may be due to the question prompt which offered examples in place of an actual definition. Consequently, we could not ascertain the extent of exposure among participants who indicated that they were employed, as it has been demonstrated that infection rates and death rates were disproportionately high among essential workers in Toronto during the pandemic [27].

Throughout the survey, participants with lower incomes, lower education backgrounds, poorer health literacy, as well as those who were non-white, and those that did not speak English as a first language, were less likely to answer questions. This may reflect an increase in non-response bias, as low-income households have witnessed a sharp drop in survey participation during the pandemic [28]. The direction in which this could bias our results is unclear, as racial minorities with cancer may be more willing to receive the vaccine, but existing hesitancy among other groups may dissipate over time as the safety of vaccines is clearly demonstrated [29]. The exclusion of patients with cancer from vaccine clinical trials could also have had the effect of encouraging hesitancy [2]. 

The lack of responses among those participants may point to confusion on their part. Scholars have pointed to the existence of a “misinfodemic” following the emergence of COVID-19, which has been compounded by the fact that factual information is inaccessible for much of the public [30]. The information that is available may be oversimplified and severely lacking in nuance [31]. This underscores the need to engage those sections of the population that are at risk for low health literacy to determine how they are coping with cancer amid the pandemic. Those with low socio-economic status would have already been underserved by the cancer system, a process further exacerbated by the pandemic [32]. This should also be targeted to patients that had difficulty coping, including younger patients and women [33].

### Limitations 

Our findings are limited by our well-educated and wealthy population. Pandemic fatigue may also have contributed to a lower response rate compared to the prior version of this study. It is possible that as the pandemic has worn on, patients have adapted to its restrictions or have adjusted their expectations to the reality of the disease. Patients with cancer have reported increased resilience during the pandemic [16]. Participants who were no longer in active treatment were better represented among our participants, and recruitment at an earlier stage in the cancer journey may have produced different results. 

## 5. Conclusions

This paper provides another snapshot of the state of patients with cancer during the second year of the COVID-19 pandemic. It demonstrates increased optimism among participants in 2021, relative to 2020, that the virus can be controlled and would not disrupt their cancer treatment. These patients continue to demonstrate strong adherence to infection control measures and are knowledgeable about the disease. While vaccine uptake was high, there appear to be significant gaps in information associated with racial minorities, low-income patients, and likely those with low health literacy. Future studies must address the needs of the most disadvantaged patients, who are part of a larger segment of society that has been disproportionately affected by the pandemic. 

## Figures and Tables

**Table 1:** Demographics.

Variables	*N* (%)
Gender	
Male	839 (49)
Female	861 (51)
Other	4 (0)
Missing	56
Age	
Mean (SD)	61.8 (13.9)
Median (Min, Max)	64 (19,100)
Missing	83
Sexuality	
Heterosexual	1534 (91)
Other	160 (9)
Missing	66
Country of Birth	
Canada	1046 (62)
Other	654 (38)
Missing	60
First Language	
English	1298 (76)
Other	401 (24)
Missing	61
Language Spoken at Home	
English	1452 (86)
Other	242 (14)
Missing	66
Understand Health Information in English	
Yes	1655 (97)
No	49 (3)
Missing	56
Comfort Filling Out Medical Forms	
Yes	1587 (94)
No	105 (6)
Missing	68
Race	
White/Caucasian/European	1296 (76)
Other	415 (24)
Missing	49
Highest Level of Education Completed	
Low	263 (16)
Medium	1051 (62)
High	382 (23)
Missing	64
Annual Household Income	
Less than USD 40,000	223 (17)
USD 40,000–59,999	173 (13)
USD 60,000–99,999	329 (25)
More than USD 100,000	589 (45
Missing	46
Main Work-Related Activity	
Working (part time or full-time)	684 (40)
Retired	20 (1)
Other	1005 (59)
Missing	51
Marital Status	
Married/common law	223 (13)
Other	1484 (87)
Missing	53
Living Arrangements	
Alone	336 (20)
Not alone	1366 (80)
Missing	58
Do you live with someone whose job puts them in contact with others?	
Yes	531 (39)
No	780 (58)
Not sure	37 (3
Missing	412
Are you an essential worker?	
Yes	487 (74)
No	175 (26)
Missing	1098
If you live with someone whose job puts them in contact with others, are they vaccinated?	
Yes—fully vaccinated	484 (91)
Yes—partially vaccinated	15 (3)
No	16 (3)
Not sure	15 (3)
Missing	1230
Cancer Type	
Blood cancer	308 (19)
Other	1288 (81)
Missing	164
Treatment Stage	
In treatment	281 (17)
Not in treatment	377 (23)
Follow-up	1003 (60)
Missing	99
How has COVID-19 changed your appointments?	
Made virtual	948 (59)
No change	667 (41)
Missing	145

**Table 2:** Treatment Impact of COVID-19.

Variables	*N* “Yes”
Delayed by less than 2 weeks	
No	1634 (98)
Yes	27 (2)
Missing	99
Delayed by more than two weeks but less than 3 months	
No	1575 (95)
Yes	86 (5)
Missing	99
Delayed by more than 3 months	
No	1641 (99)
Yes	20 (1)
Missing	99
Delayed by more than 3 months but less than 6 months	
No	1642 (99)
Yes	19 (1)
Missing	99
Delayed by more than 6 months	
No	1641 (99)
Yes	20(1)
Missing	99
Delayed and I do not know when it will be rescheduled	
No	1656 (100)
Yes	5 (0)
Missing	99
No change; my treatments were carried out as planned	
No	622 (37)
Yes	1039 (63)
Missing	99

**Table 3:** Cancer worry.

I Am Worried/Afraid That…	*N* (%)					
	Strongly Disagree	Disagree	Neutral	Agree	Strongly Agree	Missing
The COVID-19 pandemic and the response to it will make it hard for me to get cancer care in the future.	267 (16)	401 (25)	472 (29)	367 (23)	120 (7)	133
I will experience complications with my current cancer treatment because of the COVID-19 pandemic.	66 (21)	95 (30)	106 (33)	35 (11)	16 (5)	1442
My cancer will return and not be detected or managed properly because of the COVID-19 pandemic.	232 (18)	386 (30)	339 (27)	221 (17)	89 (7)	493
I will get COVID-19 by coming to the cancer centre.	358 (31)	440 (38)	240 (21)	101 (9)	31 (3)	590

**Table 4:** Source of information.

	Never/ Very Poor	Rarely/ Poor	Sometimes/ Neutral	Often/ Good/	Always/ Excellent	Missing
Television stations						
Usage	156 (10)	197 (12)	347 (22)	508 (32)	380 (24)	172
Quality/trustworthiness	52 (3)	130 (9)	385 (26)	730 (49)	192 (13)	271
Daily or weekly print newspapers						
Usage	547 (37)	258 (17)	279 (19)	235 (16)	166 (11)	275
Quality/trustworthiness	41 (4)	73 (6)	363 (32)	533 (47)	129 (11)	621
Websites or online news pages						
Usage	130 (8)	157 (10)	459 (29)	489 (31)	326 (21)	199
Quality/trustworthiness	50 (3)	133 (9)	569 (40)	585 (41)	100 (7)	323
Public health department and press releases						
Usage	86 (5)	143 (9)	460 (29)	519 (33)	370 (23)	182
Quality/trustworthiness	26 (2)	33 (2)	202 (14)	694 (47)	534 (36)	271
Conversations with friends and family						
Usage	85 (5)	302 (19)	651 (41)	414 (26)	145 (9)	163
Quality/trustworthiness	56 (4)	172 (12)	761 (52)	429 (29)	53 (4)	289
Conversations with work colleagues						
Usage	394 (32)	297 (24)	344 (28)	162 (13)	52 (4)	511
Quality/trustworthiness	85 (9)	139 (14)	520 (54)	189 (20)	28 (3)	799
Journal articles						
Usage	432 (30)	367 (25)	446 (31)	166 (11)	43 (3)	306
Quality/trustworthiness	31 (3)	46 (4)	393 (35)	452 (40)	196 (18)	642
Social media						
Usage	665 (44)	305 (20)	320 (21)	150 (10)	86 (6)	234
Quality/trustworthiness	353 (30)	319 (27)	323 (28)	150 (13)	24 (2)	591
Search engines						
Usage	273 (18)	300 (19)	578 (37)	306 (20)	100 (6)	203
Quality/trustworthiness	86 (6)	167 (12)	605 (45)	443 (33)	58 (4)	401
Radio stations						
Usage	380 (25)	321 (21)	468 (31)	260 (17)	103 (7)	228
Quality/trustworthiness	63 (5)	99 (8)	482 (39)	501 (40)	95 (8)	520
Cancer centre resources						
Usage	432 (28)	448 (29)	437 (28)	148 (10)	78 (5)	217
Quality/trustworthiness	20 (2)	34 (3)	261 (22)	476 (41)	373 (32)	596

**Table 5:** Psychological impact of COVID-19.

Question	*N* (%)						
	Strongly Disagree	Disagree	Neutral	Agree	Strongly Agree	Does Not Apply	Missing
It has been difficult to focus on tasks because of concerns about COVID-19	214 (14)	487 (31)	312 (20)	436 (28)	104 (7)	21 (1)	186
It has been difficult for me to sleep because of concerns about COVID-19	382 (24)	570 (36)	331 (21)	220 (14)	49 (3)	25 (2)	183
I have had fears about getting COVID-19	128 (8)	256 (16)	308 (20)	641 (41)	223 (14)	18 (1)	186
I have had fears of family/loved ones getting COVID-19	62 (4)	149 (9)	183 (12)	778 (49)	384 (24)	18 (1)	186
I have had fears of friends getting COVID-19	78 (5)	176 (11)	306 (19)	785 (50)	206 (13)	19 (1)	190
I have felt socially isolated from friends and family because of COVID-19	75 (5)	195 (12)	200 (13)	657 (42)	426 (27)	17 (1)	190
I have felt angry and irritable because of COVID-19	202 (13)	388 (25)	369 (23)	431 (27)	162 (10)	21 (1)	187
I have felt anxious about financial concerns because of COVID-19	263 (17)	462 (29)	311 (20)	281 (18)	192 (12)	64 (4)	187

**Table 6:** Knowledge about COVID-19.

Question	*N* (%)			
	True	False	I Do Not Know	Missing
Symptoms of COVID-19 include fever, fatigue, dry cough, and muscle pain	1430 (93)	36 (2)	71 (5)	223
Unlike the common cold, stuffy nose, runny nose, and sneezing are less common in people who have COVID-19.	573 (37)	600 (39)	364 (24)	223
Right now, there is no cure for COVID-19, but catching symptoms early and getting treatment can help patients recover from the virus.	1250 (81)	166 (11)	123 (8)	221
Not all people with COVID-19 will develop to severe cases. Seniors and people with chronic illnesses are more likely to be severe cases.	1451 (94)	51 (3)	40 (3)	218
Eating or touching wild animals can cause you to become sick with the COVID-19 virus.	87 (6)	1132 (73)	322 (21)	219
People with COVID-19 cannot give the virus to others when they do not have a fever.	52 (3)	1389 (90)	95 (6)	224
The COVID-19 virus spreads via respiratory droplets through coughing, sneezing or intimate contact.	1493 (97)	19 (1)	22 (1)	226
Wearing a medical mask can help prevent the COVID-19 virus from spreading.	1494 (97)	22 (1)	25 (2)	219
Children and young adults do not have to take measures to prevent the spread of the COVID-19 virus.	28 (2)	1480 (96)	32 (2)	220
To prevent the spread of COVID-19, people should limit (stop) going to crowded places and limit taking public transportation.	1319 (86)	148 (10)	73 (5)	220
Isolation and treatment of people with COVID-19 are ways to slow down the spread of the virus.	1474 (96)	26 (2)	40 (3)	220
People who have contact with someone who has the COVID-19 virus should be isolated in a safe place for at least 14 days.	1443 (94)	55 (4)	44 (3)	218
The incubation period of COVID-19 can be up to 14 days.	1399 (91)	36 (2)	106 (7)	219
People with cancer have to be more careful than people to protect themselves against COVID-19.	1394 (90)	51 (3)	97 (6)	218

**Table 7:** COVID-19 preventative behaviours.

Actions	*N* (%)			
	Yes	No	Does Not Apply	Missing
Hand washing for 20 s	1493 (98)	32 (2)	5 (0)	230
Did not touch your eyes, nose, and mouth with unwashed hands	1362 (89)	159 (10)	10 (1)	229
Used disinfectants to clean your hands	1496 (98)	33 (2)	4 (0)	227
Stayed home when you were sick or had a cold	1153 (75)	8 (1)	370 (24)	229
Did not go near someone who was sick or had a cold	1342 (88)	82 (5)	106 (7)	230
Wore personal protective equipment when leaving home	1501 (98)	29 (2)	2 (0)	228
Only made essential trips outside of the home	1357 (89)	160 (10)	16 (1)	227
Did not go to crowded places	1398 (91)	118 (8)	15 (1)	229
Practiced social distancing as much as possible	1505 (98)	22 (1)	5 (0)	228
Self-quarantined	699 (46)	312 (20)	520 (34)	229

**Table 8:** Attitudes about COVID-19.

Question	*N* (%)					
	Strongly Agree	Agree	Neutral	Disagree	Strongly Disagree	Missing
Do you think the COVID-19 pandemic can be successfully controlled?	316 (21)	889 (59)	231 (15)	72 (5)	0 (0)	252
Do you think that Princess Margaret Cancer Centre has done a good job of responding to the COVID-19 pandemic?	672 (45)	697 (46)	127 (8)	11 (1)	0 (0)	253
As a person affected by cancer, do you feel confident that you know what to do to protect yourself from COVID-19?	648 (43)	781 (52)	70 (5)	13 (1)	0 (0)	248

**Table 9:** Vaccinations.

Question	*N* (%)
Do you believe that the COVID-19 vaccines will help control the spread of COVID-19?	
Yes	40 (3)
No	1407
I do not know	59 (4)
Missing	254
Have you had at least one dose of the vaccine?	
No	46 (3)
Yes	1464 (97)
Missing	250
If you have not had the vaccine, do you plan on doing so?	
No	23 (51)
Yes	4 (9)
Undecided	18 (40)
Missing	1715
What is your motivation for getting the vaccine or wanting to get the vaccine? (Select all that apply)	
Protect my health	
No	185 (12)
Yes	1365 (88)
Missing	210
Protect health of family/friends	
No	305 (20)
Yes	1245 (80)
Missing	210
Protect health of co-workers	
No	1012 (65)
Yes	538 (35)
Missing	210
Protect health of community	
No	460 (30)
Yes	1090 (70)
Missing	210
To get back to work/school	
No	1188 (77)
Yes	362 (23)
Missing	210
To resume social activities	
No	636 (41)
Yes	914 (59)
Missing	210
To resume travel	
No	780 (50)
Yes	770 (50)
Missing	210
Because others encouraged me to get the vaccine	
No	1443 (93)
Yes	107 (7)
Missing	210
Not sure	
No	1532 (99)
Yes	18 (1)
Missing	210

**Table 10:** COVID-19 and discrimination.

Question	*N* (%)
Do you think that you should avoid people from countries where COVID-19 case numbers are/deaths are high (for example Brazil or India)?	
Yes	727 (48)
No	465 (31)
I do not know	249 (17)
I prefer not to say	68 (5)
Missing	251
Have you seen, heard, or experienced any incidents of discrimination related to COVID-19?	
Yes	338 (22)
No	1170 (78)
Missing	252
What was your role?	
You were the target	30 (9)
You witnessed it	137 (41)
You supported someone who experienced it	64 (19)
Other	105 (31)

**Table 11:** Multivariate analysis.

	*N*	Adjusted Estimate (95% CI)	*p*-Value	Holm Adj *p*
**Model 1. Psychological Impact**				
Ethnicity	1570		<0.001	**0.001**
White	1208	Reference		
Other	362	3.30 (1.30, 5.30)		
Use of Journal Articles	1412		0.001	**0.002**
Never	416	Reference		
Rarely	353	2.26 (−6 × 10^−3^, 4.53)	0.032	0.051
Sometimes	436	3.65 (1.50, 5.80)	<0.001	<0.001
Often	165	4.58 (1.68, 7.47)	0.001	0.002
Always	42	5.46 (0.39, 10.52)	0.035	0.035

**Table 12:** Multivariate analysis.

	*N*	Adjusted Odds Ratio (95% CI)	*p*-Value	Holm Adj *p*
**Model 1. Vaccine Uptake**				
Age	1475	1.03 (1.00, 1.06)	<0.001	**0.035**
Income	1155		<0.001	**0.001**
Less than USD 400,00	184	Reference		
USD 40,000–59,999	148	6.00 (1.26, 28.54)	0.019	0.024
USD 60,000–99,999	296	4.48 (1.48, 13.58)	0.005	0.008
More than USD 100,000	527	6.52 (2.41, 17.66)	<0.001	<0.001
Television stations usage	1478		<0.001	0.07
Never	143	Reference		
Rarely	182	2.07 (0.51, 8.36)	0.08	0.3
Sometimes	323	1.04 (0.34, 3.20)	0.38	0.95
Often	475	3.49 (0.90, 13.62)	0.004	0.072
Always	355	5.35 (0.96, 29.64)	0.001	0.055
Public health department and press releases usage	1467		<0.001	**0.038**
Never	76	Reference		
Rarely	134	0.79 (0.19, 3.18)	0.45	0.74
Sometimes	420	3.29 (0.84, 12.94)	0.005	0.088
Often	488	3.30 (0.83, 13.13)	<0.001	0.09
Always	349	5.17 (0.84, 31.69)	<0.001	0.076
**Model 2. I am worried that the COVID-19 pandemic and the response to it will make it hard for me to get cancer care in the future**				
Age	1590	0.99 (0.98, 1.00)	<0.001	**0.004**
Public health department and press releases usage	1466		0.01	**0.002**
Never	25	Reference		
Rarely	32	1.06 (0.23, 4.79)	0.82	0.94
Sometimes	199	0.44 (0.14, 1.42)	0.52	0.17
Often	683	0.33 (0.11, 1.04)	0.16	0.06
Always	527	0.27 (0.09, 0.85)	0.11	0.03
**Model 3. I fear that my cancer will return and not be detected or managed properly because of the COVID-19 pandemic**				
Age	1239	0.98 (0.98, 0.99)	<0.001	**<0.001**
Public health department and press releases usage	1151			
Never	19	Reference		
Rarely	22	0.74 (0.21, 2.59)	0.43	0.64
Sometimes	159	0.54 (0.21, 1.37)	0.28	0.19
Often	529	0.45 (0.18, 1.09)	0.09	0.08
Always	422	0.34 (0.14, 0.85)	0.03	0.02
**Model 4. I am afraid of getting COVID-19 by coming to the cancer centre**				
Ethnicity	1167			
White	895	Reference	<0.001	**0.03**
Other	272	1.53 (1.05, 2.21)		
Television stations usage	1124		0.002	**0.04**
Never	102	Reference		
Rarely	132	0.63 (0.35, 1.13)	0.16	0.12
Sometimes	252	0.81 (0.48, 1.37)	0.91	0.44
Often	355	0.78 (0.47, 1.30)	1.00	0.35
Always	283	1.24 (0.73, 2.09)	0.07	0.43
Public health department and press releases usage	1057		0.002	**0.02**
Never	16	Reference		
Rarely	20	3.29 (0.45, 24.26)	0.003	0.24
Sometimes	130	6.10 (1.07, 34.87)	0.004	0.04
Often	493	5.26 (0.95, 28.99)	0.01	0.06
Always	398	3.73 (0.68, 20.57)	0.03	0.13
**Model 5. Daily or weekly print newspapers usage**				
Age	1553	1.02 (1.01, 1.03)	<0.001	**<0.001**
Education	1572		<0.001	**<0.001**
Low		Reference		
Medium		0.75 (0.56, 0.99)	<0.001	**0.043**
High		0.54 (0.38, 0.74)	<0.001	<0.001
Television stations usage	1468		<0.001	<0.001
Never	50	Reference		
Rarely	125	2.61 (1.28, 5.33)	0.002	**0.008**
Sometimes	379	4.56 (2.33, 8.90)	<0.001	<0.001
Often	724	14.49 (7.45, 28.20)	<0.001	<0.001
Always	190	47.75 (23.11, 98.68)	<0.001	<0.001
Age	1455	1.05 (1.04, 1.07)	<0.001	**<0.001**
Education	1470		<0.001	**0.028**
Low	218	Reference		
Medium	907	1.38 (0.87, 2.18)	0.065	0.17
High	345	1.98 (1.17, 3.35)	<0.001	0.011
Daily or weekly print newspapers Perceived Quality	1085		<0.001	**<0.001**
Very poor	37	Reference		
Poor	70	11.54 (2.28, 58.51)	<0.001	0.003
Neutral	345	20.46 (4.50, 93.01)	<0.001	<0.001
Good	510	63.11 (13.88, 286.92)	<0.001	<0.001
Excellent	123	228.97 (47.41, 1.1 × 10^3^)	<0.001	<0.001
Age	1529	0.98 (0.97, 0.99)	<0.001	**<0.001**
Education	1546		<0.001	**<0.001**
Low	232	Reference		
Medium	960	1.61 (1.12, 2.31)	<0.001	0.01
High	354	2.28 (1.50, 3.47)	<0.001	<0.001
Websites or online news pages usage	1406		<0.001	**<0.001**
Never	47	Reference		
Rarely	129	3.40 (1.50, 7.67)	<0.001	0.003
Sometimes	555	4.52 (2.14, 9.54)	<0.001	<0.001
Often	575	14.34 (6.74, 30.54)	<0.001	<0.001
Always	100	34.96 (14.76, 82.77)	<0.001	<0.001
**Model 6. Public Health Department and Press Releases**				
Public health department and press releases perceived quality	1462		<0.001	**<0.001**
Very poor	25	Reference		
Poor	32	9.20 (3.13, 27.09)	<0.001	<0.001
Neutral	194	11.16 (4.52, 27.55)	<0.001	<0.001
Good	681	26.07 (10.66, 63.73)	<0.001	<0.001
Excellent	530	68.64 (27.71, 170.06)	<0.001	<0.001
**Model 7. Conversations with friends and family**				
Language Spoken	1575		<0.001	**<0.001**
English	1362	Reference		
Other	213	1.82 (1.28, 2.60)		
Conversations with friends and family perceived quality	1575		<0.001	**<0.001**
Very poor	55	Reference		
Poor	168	5.42 (2.81, 10.44)	<0.001	<0.001
Neutral	758	24.89 (13.29, 46.61)	<0.001	<0.001
Good	423	95.60 (49.84, 183.38)	<0.001	<0.001
Excellent	53	309.87 (135.65, 707.84)	<0.001	<0.001
**Model 8. Conversations with work colleagues**				
Employment	1244		<0.001	**<0.001**
Working (part-time or full-time)	618	Reference		
Retired	16	5.80 (1.73, 19.40)	0.025	**0.004**
Other	610	0.33 (0.24, 0.46)	<0.001	<0.001
Q16 Contact Others	994		<0.001	**0.029**
No	546	Reference		
Yes	422	1.46 (1.09, 1.95)	<0.001	0.01
Not sure	26	0.93 (0.42, 2.06)	0.006	0.86
Conversations with work colleagues perceived quality	925		<0.001	<0.001
Very poor	84	Reference		
Poor	135	5.57 (2.89, 10.76)	<0.001	<0.001
Neutral	500	15.10 (8.25, 27.64)	<0.001	<0.001
Good	178	90.14 (45.21, 179.72)	<0.001	<0.001
Excellent	28	395.60 (136.54, 1.1 × 10^3^)	<0.001	<0.001
**Model 9. Social Media usage**				
Age	1494	0.96 (0.95, 0.98)	<0.001	**<0.001**
Social media perceived quality	1143		<0.001	**<0.001**
Very poor	344	Reference		
Poor	315	8.19 (5.36, 12.50)	<0.001	<0.001
Neutral	313	21.54 (13.49, 34.40)	<0.001	<0.001
Good	148	100.90 (55.71, 182.74)	<0.001	<0.001
Excellent	23	479.18 (142.20, 1.6 × 10^3^)	<0.001	<0.001
**Model 10. Search engine usage**				
Age	1525	0.98 (0.97, 0.99)	<0.001	**<0.001**
Country Born	1540		<0.001	**0.004**
Canada	977	Reference		
Other	563	1.43 (1.12, 1.84)		
Search engines perceived quality	1342		<0.001	**<0.001**
Very poor	82	Reference		
Poor	164	8.02 (4.54, 14.16)	<0.001	<0.001
Neutral	598	26.65 (15.68, 45.29)	<0.001	<0.001
Good	440	108.02 (61.89, 188.51)	<0.001	<0.001
Excellent	58	215.48 (101.66, 456.71)	<0.001	<0.001
**Model 11. Radio Station usage**				
Radio stations perceived quality	1209		<0.001	**<0.001**
Very poor	60	Reference		
Poor	95	2.73 (1.44, 5.18)	0.002	0.002
Neutral	471	8.96 (5.06, 15.86)	<0.001	<0.001
Good	489	36.86 (20.51, 66.26)	<0.001	<0.001
Excellent	94	174.18 (86.79, 349.56)	<0.001	<0.001
**Model 12. Cancer Centre resources usage**				
Cancer centre resources perceived quality	1140		<0.001	**<0.001**
Very poor	18	Reference		
Poor	33	6.79 (1.69, 27.25)	0.008	0.007
Neutral	255	13.33 (3.79, 46.93)	<0.001	<0.001
Good	466	39.57 (11.28, 138.83)	<0.001	<0.001
Excellent	368	67.36 (19.06, 238.07)	<0.001	<0.001

## Data Availability

The data presented in this study are available in this article (and supplementary material).

## Supplementary Materials

The following supporting information can be downloaded at: https://www.mdpi.com/article/10.3390/curroncol29110701/s1. The following supplementary material is attached as part of this submission: “Supplementary File (survey)”.
