# Supplementary material for: COVID-19 and Cancer Patients in the Second Year of the Pandemic: Investigating Treatment Impact, Information Sources, and COVID-19-Related Knowledge, Attitudes and Practices
Source: Curr Oncol. 2022 Nov 18;29(11):8917–36. doi: 10.3390/curroncol29110701 (PMC9689213; doi:10.3390/curroncol29110701)
Supplement: Supplementary file 1 [file curroncol-29-00701-s001.zip › curroncol-1986648-supplementary.pdf]

Supplementary File (survey)

Section 1. About You

Please respond to the following questions about you:

1. What is your age? __________

2. What is your gender
   - Male
   - Female
   - Trans male
   - Trans female
   - Intersex
   - Non-Binary
   - Other (Please list): ________________________

3. What country were you born in?
   - Canada
   - Other Country (Please list): ________________________

4. What is your first language?
   - English
   - Other: _______________________________

5. What language do you speak most often at home?
   - English
   - Other: ______________________

6. Do you understand health information in English?
   - All the time
   - Most of the time
   - Some of the time
   - Never

7. Do you feel comfortable filling out medical forms on your own?
   - Yes
   - No

8. What is your ethnicity?
   - Indigenous (Inuit, Métis, First Nations)
   - Arab/West Asian (Armenian, Egyptian, Iranian, Lebanese, Moroccan)
   - Black/African (African-Canadian, Haitian, Jamaican, Somali)
   - East Asian (Chinese, Japanese, Korean)

- Latin American/Latino
- South Asian (Indian, Pakistani, Bangladeshi)
- South East Asian (Filipino, Indonesian, Malaysian)
- White/Caucasian/European
- I prefer not to answer
- Other (please list): ________________________

9. What is the highest level of schooling you completed?
   - Grade school
   - Some high school
   - High school
   - Some college/university
   - College/University
   - Postgraduate school
   - Other (please list): ________________________

10. What was your household income in the last year?
    - Less than $40,000
    - $40,000-$59,999
    - $60,000-$79,999
    - $80,000-$99,999
    - More than $100,000
    - I prefer not to say

11. What is your marital status?
    - Single, never married
    - ☐ Married, Common-law
    - ☐ Separated
    - ☐ Divorced
    - ☐ Widowed
    - ☐ Other: ________________

12. Do you consider yourself to be:
    - ☐ Heterosexual or straight
    - ☐ Gay or lesbian
    - ☐ Bisexual
    - ☐ Prefer not to answer
    - ☐ Other: __________________________

13. What is your main work-related activity?
    - Working (part-time or full-time)
    - Student
    - Homemaker
    - Getting disability payment

- Retired
- Unemployed
- Other (please list): ________________________

14. Are you an essential worker (e.g. grocery store worker, bus driver, healthcare worker, etc.)?
    - ☐ Yes
    - ☐ No

15. What are your living arrangements?
    I live:
    - I live alone
    - I do not live alone

16. Do you live with someone whose job puts them in contact with others?
    - ☐ Yes
    - ☐ No
    - ☐ Not sure

17. If you live with someone whose job puts them in contact with others, are they vaccinated?
    - ☐ Yes – fully vaccinated
    - ☐ Yes – partially vaccinated
    - ☐ No
    - ☐ Not sure

18. Enter the first three digits of your postal code (We want to know how far you live from Princess Margaret Cancer Centre)? ________________________

19. What is your cancer type?

- ☐ Blood (Leukemia, Lymphoma, Multiple Myeloma)
- ☐ Breast Cancer
- ☐ Eye
- ☐ Gastrointestinal (Ampullary, Appendix, Anus, Colon, Esophageal, Gall Bladder, Liver, Pancreatic, Stomach, Carcinoid)
- ☐ Genitourinary (Bladder, Kidney, Penile, Prostate, Testicular)
- ☐ Gynecologic (Cervical, Endometrial or Uterine, Ovarian, Vaginal, Vulvar, Gestational Trophoblastic)
- ☐ Head and Neck (Mouth, Nose, Throat
- ☐ Lung
- ☐ Sarcoma (Bone, Cartilage, Fat, Muscle)
- ☐ Skin and Melanoma
- ☐ I do not know
- ☐ I have not been diagnosed

Other: _______________________________

20. Which of these best describes where you are with your cancer?
    - Newly diagnosed and have not started treatment yet
    - Newly diagnosed and getting treatment now
    - Recently finished treatment (less than 3 months after treatment)
    - Short-term follow-up (less than one year after treatment)
    - Long-term follow-up (more than one year after treatment)
    - Remission (cancer is gone) and monitoring
    - Recurrent cancer (cancer came back) and started treatment
    - Recently finished treatment for recurrent cancer

Section 2. Treatment Impact of COVID-19
Please respond to the following questions about how COVID-19 has affected your cancer treatment since social distancing measures were put in place by the province:
Check all that apply.

1. If you are in active treatment, how has COVID-19 affected your treatment schedule?
    - Delayed by less than two weeks
    - Delayed by more than two weeks, but less than 3 months
    - Delayed by more than 3 months
    - Delayed by more than 3 months, but less than 6 months
    - Delayed by more than 6 months
    - Delayed and I do not know when it will be rescheduled
    - No change; my treatments carried out as planned

2. How has COVID-19 affected your appointments?
    - In-person visits changed to phone or video
    - No change; my appointments were carried out as planned

3. What part of your cancer care or treatment was delayed? **(check all that apply)**
    - In-person appointments with your oncologist (cancer doctor)
    - Access to imaging services to see if your cancer has grown or returned (e.g. MRI)
    - Access to supportive services (e.g. physical therapy, mental health care)
    - Access to surgical procedures (e.g. chemotherapy, immunotherapy)
    - Does not apply; my care was not delayed

4. I am worried that the COVID-19 pandemic and the response to it will make it hard for me to get cancer care in the future.
    - Strongly Disagree
    - Disagree
    - Neutral
    - Agree

- Strongly Agree

5. [If in active treatment] I am worried that I will experience complications with my current cancer treatment because of the COVID-19 pandemic.
    - Strongly Disagree
    - Disagree
    - Neutral
    - Agree
    - Strongly Agree

6. [if agree/strongly agree] What treatment complications are you worried about: ________________

7. [if in follow-up] I fear my cancer will return and not be detected or managed properly because of the COVID-19 pandemic.
    - Strongly Disagree
    - Disagree
    - Neutral
    - Agree
    - Strongly Agree

8. [if treatment not delayed] I am afraid of getting COVID-19 by coming to the Cancer Centre?
    - Strongly Disagree
    - Disagree
    - Neutral
    - Agree
    - Strongly Agree

Section 3. Information about COVID-19
Please check one box per row to show how often you use information from each source to stay informed about COVID-19:

	Never	Rarely	Sometimes	Often	Always	Does not Apply
1. Television stations	☐	☐	☐	☐	☐	☐
2. Daily or weekly print newspapers	☐	☐	☐	☐	☐	☐
3. Websites or online news pages	☐	☐	☐	☐	☐	☐

	Never	Rarely	Sometimes	Often	Always	Does not Apply
4. Public health department and press releases (in the news or other sources)	☐	☐	☐	☐	☐	☐
5. Conversations with friends and family (in-person, telephone, video call or email)	☐	☐	☐	☐	☐	☐
6. Conversations with work colleagues (in-person, telephone, video call or email)	☐	☐	☐	☐	☐	☐
7. Journal Articles	☐	☐	☐	☐	☐	☐
8. Social media (e.g. Facebook, Twitter, YouTube, WhatsApp)	☐	☐	☐	☐	☐	☐
9. Search engines (e.g. Google)	☐	☐	☐	☐	☐	☐
10. Radio stations	☐	☐	☐	☐	☐	☐
11. Cancer Centre resources (e.g. brochures, videos, website)	☐	☐	☐	☐	☐	☐
Other (please list): ______________________________						

Please check the box to show how you view the quality and trustworthiness of information about the COVID-19 from each source:

	Very Poor	Poor	Neutral	Good	Excellent	Does Not Apply
12. Television stations	☐	☐	☐	☐	☐	☐
13. Daily or weekly print newspapers	☐	☐	☐	☐	☐	☐

	Very Poor	Poor	Neutral	Good	Excellent	Does Not Apply
14. Websites or online news pages	☐	☐	☐	☐	☐	☐
15. Public health department and press releases (in the news or other sources)	☐	☐	☐	☐	☐	☐
16. Conversations with friends and family (in-person, telephone, video call or email)	☐	☐	☐	☐	☐	☐
17. Conversations with work colleagues (in-person, telephone, video call or email)	☐	☐	☐	☐	☐	☐
18. Journal Articles	☐	☐	☐	☐	☐	☐
19. Social media (e.g. Facebook, Twitter, YouTube, WhatsApp)	☐	☐	☐	☐	☐	☐
20. Search engines (e.g. Google)	☐	☐	☐	☐	☐	☐
21. Radio stations	☐	☐	☐	☐	☐	☐
22. Cancer Centre resources (e.g. brochures, videos, website)	☐	☐	☐	☐	☐	☐
Other (please list): ______________________________						

Section 4. Psychological Impact of COVID-19
Please check the box showing how much you agree with each statement:

	Strongly Disagree	Disagree	Neutral	Agree	Strongly Agree	Does not Apply
1. It has been difficult to focus on tasks because of concerns about COVID-19	☐	☐	☐	☐	☐	☐

	Strongly Disagree	Disagree	Neutral	Agree	Strongly Agree	Does not Apply
2. It has been difficult for me to sleep because of concerns about COVID-19	☐	☐	☐	☐	☐	☐
3. I have had fears about getting COVID-19	☐	☐	☐	☐	☐	☐
4. I have had fears of family/loved ones getting COVID-19	☐	☐	☐	☐	☐	☐
5. I have had fears of friends getting COVID-19	☐	☐	☐	☐	☐	☐
6. I have felt socially isolated from friends and family because of COVID-19	☐	☐	☐	☐	☐	☐
7. I have felt angry and irritable because of COVID-19	☐	☐	☐	☐	☐	☐
8. I have felt anxious about financial concerns because of COVID-19	☐	☐	☐	☐	☐	☐

Section 5. Knowledge About the COVID-19 Pandemic
This section includes true and false information about COVID-19. Due to the amount of information about COVID-19, it can be difficult to know what is true and what is not.

Please check the box to show whether you think each statement about COVID-19 is true (correct) or false (incorrect). This is not a test, it a way to assess public opinion about COVID-19:

	True	False	I Don't Know
1. Symptoms of COVID-19 include fever, fatigue (tiredness), dry cough, and muscle pain.	☐	☐	☐
2. Unlike the common cold, stuffy nose, runny nose, and sneezing are less common in people who have COVID-19.	☐	☐	☐

	True	False	I Don't Know
3. Right now, there is no cure for COVID-19, but catching symptoms early and getting treatment can help patients recover from the virus.	☐	☐	☐
4. Not all people with COVID-19 will develop to severe cases. Seniors (age 65+) and people with chronic illnesses (e.g. diabetes, heart disease), are more likely to be severe cases.	☐	☐	☐
5. Eating or touching wild animals can cause you to become sick with the COVID-19 virus.	☐	☐	☐
6. People with COVID-19 cannot give the virus to others when they do not have a fever.	☐	☐	☐
7. The COVID-19 virus spreads through the air by coughing, sneezing or intimate (close) contact.	☐	☐	☐
8. Wearing a medical mask can help prevent the COVID-19 virus from spreading.	☐	☐	☐
9. Children and young adults do not have to take measures to prevent the spread of the COVID-19 virus.	☐	☐	☐
10. To prevent the spread of COVID-19, people should limit (stop) going to crowded places and limit (stop) taking public transportation.	☐	☐	☐
11. Isolation and treatment of people with COVID-19 are ways to slow down the spread of the virus.	☐	☐	☐
12. People who have contact with someone who has the COVID-19 virus should be isolated in a safe place for at least 14 days.	☐	☐	☐
13. The incubation period (i.e. time from viral infection to developing symptoms of illness) of COVID-19 can be up to 14 days.	☐	☐	☐
14. People with cancer have to be more careful than people without cancer to protect themselves against COVID-19.	☐	☐	☐

Section 6. Actions Taken During the COVID-19 Pandemic

Please check the box to show what you have done to prevent infection from COVID-19.

	Yes	No	Does Not Apply
1. Hand washing for 20 seconds	☐	☐	☐
2. Did not touch your eyes, nose, and mouth with unwashed hands	☐	☐	☐
3. Used disinfectants (e.g. hand sanitizer) to clean your hands	☐	☐	☐
4. Stayed home when you were sick or had a cold	☐	☐	☐
5. Did not go near someone who was sick or had a cold	☐	☐	☐
6. Wore personal protective equipment (e.g. mask or gloves) when leaving home	☐	☐	☐
7. Only made essential trips outside of the home (e.g. shopping for food and other household supplies)	☐	☐	☐
8. Did not go to crowded places	☐	☐	☐
9. Practiced social distancing by staying 6-feet away from others as much as possible	☐	☐	☐
10. Self-quarantined (i.e. separated yourself from others, including those in your home)	☐	☐	☐
11. Other (please list): ______________________________			

Section 7. Attitudes About the COVID-19 Pandemic

Please respond to the following questions about your and experiences during the COVID-19 pandemic:

1. Do you think that the COVID-19 pandemic can be successfully controlled?
    - Strongly agree
    - Agree
    - Neutral

- Disagree
- Strongly Disagree

2. Do you think that Princess Margaret Cancer Centre has done a good job of responding to the COVID-19 pandemic?
    - Strongly agree
    - Agree
    - Neutral
    - Disagree
    - Strongly Disagree

3. [if disagree/strongly disagree] What could Princess Margaret do differently to improve its response to the COVID-19 pandemic? ____________________________

4. As a person affected by cancer, do you feel confident that you know what you can do to protect yourself from COVID-19?
    - Strongly agree
    - Agree
    - Neutral
    - Disagree
    - Strongly Disagree

Section 8. Vaccines

Please answer the following questions on your attitudes about the COVID-19 vaccines and your vaccination status.

1. Do you believe that the COVID-19 vaccines will help control the spread of COVID-19?
    - ☐ Yes
    - ☐ No
    - ☐ I do not know

2. Have you had at least one dose of the vaccine?
    - Yes
    - No

3. If you have had the vaccine, which one did you receive?
    - ☐ Pfizer-BioNTech
    - ☐ Moderna
    - ☐ AstraZeneca
    - ☐ Johnson & Johnson
    - ☐ I do not know
    - ☐ Other (Please specify ________)

4. If it was a 2-dose vaccine, have you had your second dose?

- ☐ Yes
- ☐ No
- ☐ N/A

5. If you have not had the vaccine, do you plan on doing so?
   - ☐ Yes
   - ☐ No
   - ☐ Undecided

6. What is your motivation for getting the vaccine or wanting to get the vaccine? (Select all that apply.)
   - ☐ Protect my health
   - ☐ Protect health of family/friends
   - ☐ Protect health of co-workers
   - ☐ Protect health of community
   - ☐ To get back to work/school
   - ☐ To resume social activities
   - ☐ To resume travel
   - ☐ Because others encouraged me to get the vaccine
   - ☐ Not sure
   - ☐ Other : ________________________

7. If you want to get the vaccine and have not yet done so, what made it difficult for you to get a COVID-19 vaccine? (Select all that apply.)
   - ☐ I could not go on my own (I have a physical limitation)
   - ☐ It is too far away
   - ☐ I did not know where to go to get vaccinated
   - ☐ I did not have transportation
   - ☐ The hours of operation do not work for my schedule
   - ☐ The waiting time was too long
   - ☐ It was hard to find or make an appointment
   - ☐ I was too busy to get vaccinated
   - ☐ It was hard to arrange for childcare
   - ☐ I did not have time off work
   - ☐ I do not have access to a computer
   - ☐ The online registration process was not easy
   - ☐ Other
   - ☐ Not sure
   - ☐ It was not difficult

8. If you are not planning on getting the vaccine, why?

9. What can the Princess Margaret Cancer Centre do to help with getting the vaccine?

Section 9. Discrimination and COVID-19
Please respond to the following questions about your experience

1. Do you think that you should avoid people from countries where COVID-19 case numbers/deaths are high (for example Brazil or India)?
    - Yes
    - No
    - I do not know
    - I prefer not to say

2. Have you seen, heard, or experienced any incidents of racism related to COVID-19?
    - Yes
    - No

3. [If Yes] What was your role?
    - You were the target
    - You witnessed it
    - You supported someone who experienced it
    - Other (please list): ________________________

4. [If Yes] Where did this occur?
    - On the street
    - On public transportation
    - In a business
    - In a grocery store
    - In a workplace
    - In a residence
    - Online
    - In a hospital or medical setting
    - In a school setting
    - Other (please list): ________________________

5. [If Yes] What type of discrimination occurred?
    - Physical assault (like: hitting, pushing, shoving, punching)
    - Barred from public services
    - Verbal harassment (for example, name calling)
    - Online harassment
    - Coughed or spat at
    - Workplace discrimination

- Barred from business
- Shunned
- Police-related
- Other (please list): ________________________

6. [If Yes] Was the situation handled or resolved?
    - Yes
    - No
    - I do not know

Section 10. Feedback
Please provide any additional feedback you may have:

1. What information or support do you need the most right now? This may or may not include information listed in the survey.

2. Please share any comments or suggestions below:

We sincerely thank you for filling out this study.

Your feedback is very valuable and will help us develop new resources to improve the services available at Princess Margaret Cancer Centre.

If you would like to receive a copy of the results of this study once it is completed, please contact the study team at: Janet.Papadakos@uhnresearch.ca.

Thank you!
